# Molecular and Serological Diagnostic Approach to Define the Microbiological Origin of Blood Culture-Negative Infective Endocarditis

**DOI:** 10.3390/pathogens11111220

**Published:** 2022-10-22

**Authors:** Amira H. El-Ashry, Khaled Saad, Ahmed A. Obiedallah, Amira Elhoufey, Hamad Ghaleb Dailah, Mohammed Salah A. Hussein

**Affiliations:** 1 Medical Microbiology & Immunology Department, Faculty of Medicine, Mansoura University, Mansoura 35516, Egypt; 2 Department of Pediatrics, Faculty of Medicine, Assiut University, Assiut 71111, Egypt; 3 Department of Internal Medicine, Faculty of Medicine, Assiut University, Assiut 71111, Egypt; 4 Department of Community Health Nursing, Faculty of Nursing, Assiut University, Assiut 71111, Egypt; 5 Department of Community Health Nursing, Alddrab University College, Jazan University, Jazan 45142, Saudi Arabia; 6 Research and Scientific Studies Unit, College of Nursing, Jazan University, Jazan 45142, Saudi Arabia; 7 Cardiovascular Medicine Department, Faculty of Medicine, Mansoura University, Mansoura 35516, Egypt

**Keywords:** broad-range PCR, sequencing, culture negative endocarditis, serology

## Abstract

Blood culture-negative infective endocarditis (BCNIE) poses a significant challenge in determining the best antibiotic regimen for this life-threatening infection, which should be treated with as specific and effective a regimen as feasible. The goal of this study was to determine the prevalence of BCNIE among definite infective endocarditis (IE) cases and to study the impact of a molecular and serological diagnostic approach in defining the microbiological origin of BCNIE. This study included 94 definite IE cases. Serum and blood samples from BCNIE patients were tested using serological, broad-range PCR, and sequencing assays. Valve tissue sections obtained from 42 operated patients were subjected to culture and molecular studies. BCNIE accounted for 63 (67%) of the cases. Of these cases, blood PCR followed by sequencing could diagnose 11 cases. Zoonotic infective endocarditis was detected in 7 (11%) patients by serology and PCR (four *Brucella*, two *Bartonella*, and one *Coxiella*). Sequencing of valve PCR bands revealed 30 positive cases. Therefore, the percentage of BCNIE with unidentified etiology was reduced from 67% to 27.7% through a combination of all diagnostic procedures utilized in our study. Blood and valve PCR and sequencing assays are valuable techniques for the etiological diagnosis of BCNIE, especially in cases with previous antibiotic therapy. However, these tests should be used as part of a larger diagnostic strategy that includes serology, microscopy, and valve culture. The use of an automated blood culture system, and proper blood culture collection before ordering antibiotics, will guide IE etiological diagnosis.

## 1. Introduction

Blood culture-negative infective endocarditis (BCNIE) still accounts for 5–69.7% of all cases of endocarditis, despite improvements in culture media and the introduction of an automated blood culture detection system [1]. The epidemiology of infective endocarditis (IE), the diagnostic criteria employed, the antibiotics given to patients before blood cultures, and the diagnostic technique used to determine an etiology all contribute to the different incidences of BCNIE in different countries [2]. BCNIE poses a significant challenge in determining the best antibiotic regimen for this life-threatening infection, which should be treated with as specific and effective a regimen as feasible. The proper diagnosis of IE-causing infections has become even more critical in light of shifting epidemiology and the increasing number of resistant strains. In addition, insufficient treatment increases the likelihood of recurrence or re-infection [3]. BCNIE is caused by two basic factors. First, in many critical clinical situations, empirical antibiotic therapy is started before the patient is diagnosed or even suspected of having IE, leading to decreased sensitivity of blood cultures taken during this period. Second, microorganisms that are difficult to grow or have yet to be successfully cultured are frequently missed by standard culture methods [4]. Thus, culture-independent molecular approaches based on amplification and subsequent direct sequencing of bacterial and fungal ribosomal sequences have been introduced in many studies in an effort to obtain an etiological diagnosis of BCNIE [5,6,7]. In the modified Duke criteria, serological testing is now included as a diagnostic criterion for IE. Antibody titers against the most prevalent pathogens in BCNIE, *Brucella melitensis*, *Coxiella burnetii*, *Chlamydia* spp., *Bartonella* spp., *Legionella pneumophila*, and *Mycoplasma pneumoniae*, are determined in a systematic manner during BCNIE testing [8]. The goal of this study was to determine the prevalence of BCNIE among definite IE cases and to study the impact of a molecular and serological diagnostic approach in defining the microbiological origin of BCNIE.

## 2. Patients and Methods

### 2.1. Study Design

The study protocol was approved by the Institutional Review Board (IRB), Faculty of Medicine, Mansoura University, IRB code number: R/21.07.1381. In accordance with the Helsinki Declaration, each patient’s informed consent was obtained.

In a prospective observational study conducted from January 2020 to July 2021, 117 patients with suspected IE who were admitted to the Cardiology Department and or the Cardiothoracic Surgery Department of a tertiary University Hospital were enrolled as established according to modified Duke criteria for case definition. Our sample size was assessed via this formula Z^2^ * P * (1 − P)/d 2 [9], where the expected prevalence of BCNIE was estimated to be 69% in a previous study conducted by Ndiaye et al. [10]. Patients moved for valve replacement surgery and had negative blood cultures were included for valve sections. Operated patients for any other causes rather than IE were excluded.

#### Data Collection

**Figure 1:**
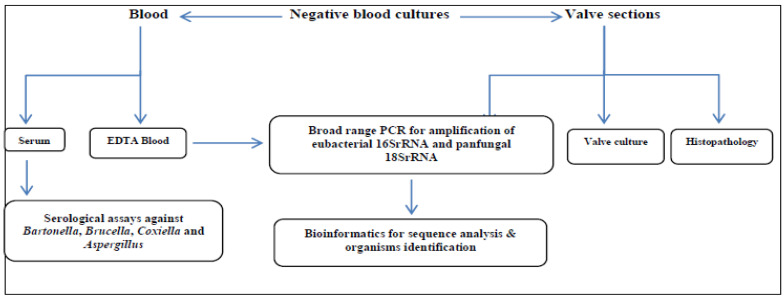
Hypothesized diagnostic approach for patients with BCNIE.

**Figure 2:**
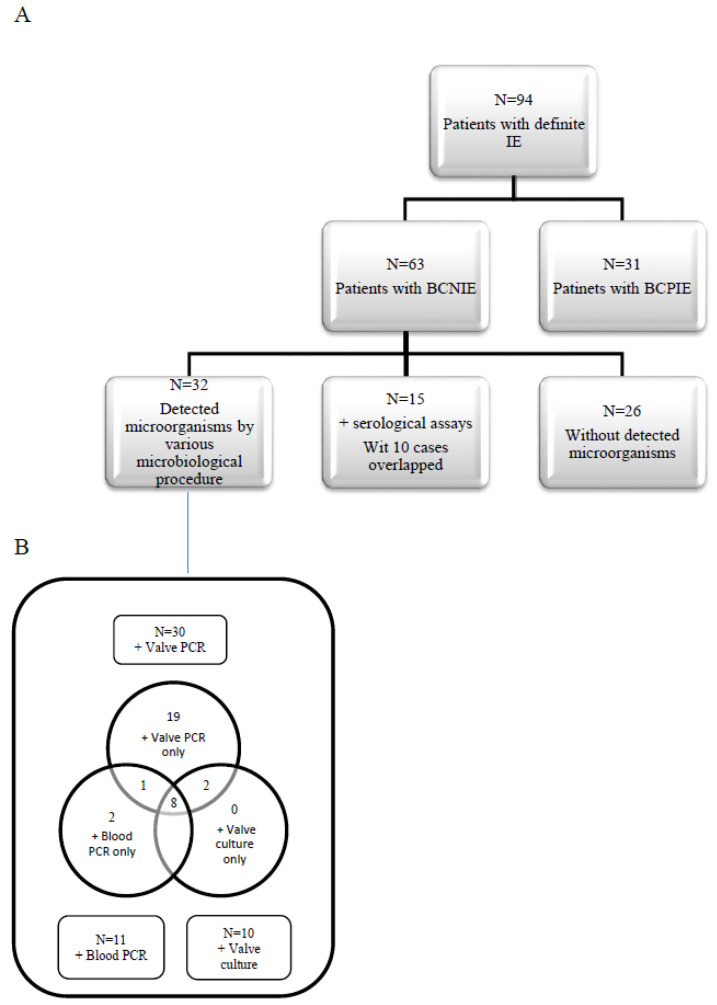
**Distribution of the determined causative agents based on the diagnostic technique.** (**A**) Flowchart of definite IE patients with detected microorganisms. (**B**) Venn diagram comparing detected microorganisms by various microbiological procedures. +: Positive results.

The following participant data were collected on a structured sheet: name, age, gender, animal contact in work, precipitating factors, history of previous attacks, clinical symptoms and consequences, affected valve(s), echocardiography findings, patient outcome, and history of antibiotic intake. Figure 1 and Figure 2 showed the hypothesized diagnostic approach for patients with BCNIE and the flowchart of definite IE patients with detected microorganisms.

### 2.2. Blood Sample Collection

Under strict aseptic technique, three blood samples were taken from all patients: three sets, each 10 mL blood drawn at least an hour apart for blood culture, all incubated aerobically and anaerobically (Oxoid Signal Blood Culture System, Thermo Scientific, Waltham, MA, USA); 3 mL blood in an Ethylenediaminetetraacetic Acid (EDTA) tube for molecular testing (Helena Laboratories, Beaumont, TX, USA); and 3 mL blood in a plain tube for serologic testing. All blood samples were rapidly transported to the microbiology laboratory, where blood bottles were incubated at 37 °C, EDTA tubes were stored at 4 °C until DNA extraction (maximum 72 h after specimen collection), and serum samples were separated, then stored at −20 °C. Bacteria were identified from blood cultures using standard microbiological techniques [11].

### 2.3. Processing of Valve Tissue Sections from BCNIE Patients

In the operating theater, excised valvular material was fragmented into three samples for further examination under sterile conditions: (1) a PCR and valve staining sample in a sterile container with no additives (the material was frozen at 20 °C within 45 min until DNA extraction); (2) a sample immersed in 4% formalin to be histopathologically examined; and (3) residual tissue transferred aseptically to the microbiology laboratory as soon as feasible for conventional microbiological techniques, including smear examination and inoculation into a bottle containing 50 mL thioglycollate broth (Oxoid, UK) for incubation at 37 °C for 3 weeks.

### 2.4. Direct Smears and Culture of Valve Sections

After grinding, a smear of each surgically excised residual valve tissue specimen was prepared and examined using Gram staining and potassium hydroxide (KOH) wet mount. Subsequent subcultures from thioglycollate broth containing heart valve tissue sections were performed in a range of microbiology media, including blood and MacConkey’s agar, chocolate agar incubated in 5% CO_2_ at 37 °C for 72 h, and Sabouraud’s dextrose agar (SDA) incubated at 25 °C for up to 21 days and checked for growth every 48 h. Morphology, Gram-stained smears, and biochemical reactions were used to identify the obtained isolates.

### 2.5. Molecular Techniques

For detection of broad-range bacterial 16S rRNA and panfungal 18S rRNA from blood and surgical materials when accessible, followed by sequencing for identification of positive cases, DNA was extracted from blood and surgical materials using the QIAamp DNA Mini Kit (Qiagen, Hilden, Germany).

16SrRNA bacterial gene amplification (matching to regions 8 to 806 of the *Escherichia coli* 16S rRNA gene) was performed using the eubacterial 16SrRNA primer set 536F 5′-CAGCAGCCGCGGTAATAC-3′ and RP2 5′-ACGGCACCTTGTTACGACTT-3′ (Bioneer, AccuOligo^®^, Oakland, CA, USA) [7], and the conserved region of the 18S (ITS-1) rRNA of fungi was amplified using the oligonucleotide panfungal primer sequences CUF 5′-TCCGTAGGTGAACCTGCGGG-3′ and CUR 5′-GCTGCGTTCTTCATCGATGC-3′ mainly targeting the V4 and V5 portions of the 18S rRNA gene, as previously designated [12].

Human β-globin housekeeping gene was amplified as an internal control by adding the primers BGF (5′-CAACTTCATCCACGTTCACC-3′) and BGR (5′ GAAGAGCCAAGGACAGGTAC-3′) to each PCR vial. *Staphylococcus aureus* (ATCC 25923), *E.* *coli* (ATCC 25922), and *Candida albicans* (ATCC 10231) were included as a positive control in each run, as was a negative control of PCR-grade water without DNA.

Amplified DNA fragments were purified from the PCR products using the Thermo Scientific™ GeneJET PCR Purification Kit according to the manufacturer’s protocol.

### 2.6. DNA Concentration and Purity

The purity and concentration of total DNA isolates in the samples were assessed by measuring the optical density (OD) at 260 nm and the OD 260/280 ratio; this analysis required only 1 µL of the sample (NanoDrop™2000 spectrophotometer, Thermo Scientific, Waltham, MA, USA).

### 2.7. DNA Cycle Sequencing

The fluorescence-based cycle sequence of PCR-amplified gene fragments was performed in the forward direction on an ABI PRISM^®^ 310 Genetic Analyzer using the forward primers 536f (5′-CAGCAGCCGCGGTAATAC-3′) and ITS-1 (5′-TCCGTAGGTGAACCTGCGGG-3′) for bacterial and fungal pathogens, respectively, using purified PCR products as the template and the ready-to-use BigDye Terminator Sequencing Kit (Applied Biosystems, Waltham, MA, USA).

### 2.8. Bioinformatics for Sequence Interpretation and Statistical Analysis

The obtained nucleotide sequences were searched in the GenBank database for comparison with those of the reference organisms using the Basic Local Alignment Search Tool (BLAST) at the National Center for Biotechnology Information (NCBI) website (http://blast.ncbi.nlm.nih.gov/Blast, accessed on 27 May 2021). The basic characteristics of the data were expressed using descriptive statistics. The results of valve PCR and valve culture were compared using Fisher’s exact test on SPSS 25.0 (IBM, Armonk, NY, USA).

### 2.9. Serological Testing

Serological assays were applied to serum samples to detect agglutinating anti-Brucella antibodies with BRUCELLACAPT (Vircell, Granada, Spain). A diagnostic *Brucella* infection titer was 1/160 [13]. An immunofluorescence assay (Vircell, Granada, Spain) was utilized for testing IgG antibodies against *Bartonella quintana, Bartonella henselae*, and *C. burnetii*. *Bartonella* and *Coxiella* endocarditis were established when the titer was 1/800 for *Bartonella* IgG and phase I *C. burnetii* IgG. *Aspergillus* galactomannan antigen (GM) was detected using the Platelia *Aspergillus* EIA (Bio-Rad, Marnes-La-Coquette, France). Patients with an *Aspergillus* antigen index of greater than 0.5 were classified as positive [14].

## 3. Results

Among 117 patients with suspected IE, 94 met the definite modified Duke criteria for endocarditis. Of these, 63 (67%) patients were diagnosed with definite BCNIE based on the combination of negative conventional blood cultures, the major echocardiographic criterion, and three minor clinical findings.

The median age of the BCNIE patients was 50 years (range 23–70 years), and their male-to-female ratio was 1.6:1. Native valve IE was found in 53 (84.1%) patients, while prosthetic valve IE was found in ten (15.9%) patients. The mitral valve was the most commonly affected, accounting for 30 (47.6%) cases, followed by the aortic and tricuspid valves, which affected 18 (28.6%) and 10 (15.9%) cases, respectively. In five (7.9%) of the cases, both the aortic and mitral valves were affected. Rheumatic heart disease was the most frequent underlying heart illness, observed in35 cases (55.6% of all BCNIE cases). Non-rheumatic valvular heart disorders were next, affecting 28.6% (18 cases). Five patients (9.5%) reported a history of intravenous drug abuse, three patients (4.8%) had intracardiac devices, and only two patients (3.2%) had congenital heart disease.

Antibiotic administration prior to blood culture collection was reported for all 63 patients with BCNIE, and both EDTA blood and serum samples were accessible. Valve tissue specimens were obtainable for 42/63 patients (66.7%) who were operated emergently or urgently for IE with valve replacement surgery. Table 1 compared positive results of sequencing of PCR positive valve section to valve culture and blood PCR. On blood collected in EDTA tubes, eight (12.7%) and three (4.8%) BCNIE blood samples were positive, respectively, in broad-range PCR for amplification of bacterial 16SrRNA and fungal 18SrRNA followed by sequencing of the amplified product (Table 2). By incorporating the blood PCR results, the percentage of BCNIE patients with no identified cause declined from 67% (63/94) to 55.3% (52/94).

According to the classification guidelines for IE by Voldstedlund et al. [15], histopathological examination of surgically removed valve tissue sections revealed the presence of typical IE in 33/42 (78.6%) of patients. In two cases, dichotomously branched septate fungal hyphae were detected in valve tissue sections stained with hematoxylin and eosin.

Valve culture was positive in 10 of the 42 valve tissue sections analyzed, resulting in a sensitivity of 23.8%, and valve Gram staining was positive in 5 of the 42 valve tissues analyzed for the existence of an organism; thus, the sensitivity was 11.9%, all of which were valve culture concordant positive. Only one valve tissue specimen was positive for *Aspergillus flavus* on KOH wet mount and SDA culture.

Valve tissue PCR using 16SrRNA primers yielded positive results in 26 of 42 (61.9%) valve sections, while broad-range fungal PCR utilizing primers for 18SrRNA yielded positive results in four-valve sections (9.5%), reducing the percentage of BCNIE from 67% to 35.1% (33/94).

Valve PCR was found to have a significant diagnostic advantage over valve culture in the ability to detect microorganisms (30/42 vs. 10/42; *X*^2^ = 5.25, *p* < 0.02).

In total, 9 of the 42 patients with positive valve section PCR also had positive blood PCR sequencing results, with 100% concordant species identified. Moreover, 2 of the 21 patients without surgical treatment were diagnosed as having *A. flavus* and *S. aureus* on blood PCR.

Four patients were found to have a positive *Brucella* serologic test with agglutinating anti-Brucella antibodies titers ≥ 1/160. Positive *B. henselae* endocarditis was detected in two patients with an IgG titer to *B. henselae* ≥ 1:800. One patient was diagnosed with chronic Q fever endocarditis (IgG titer to phase I *C. burnetii* > 1:800). Thus, the results of all serological tests performed on BCNIE serum specimens diagnosed zoonotic IE in 7/63 (11%) of patients, lowering the percentage of BCNIE with no known etiology to 59.6% (65/94).

Blood and valve tissue PCR correctly identified three of four *Brucella* cases and one of two *Bartonella* spp. cases but was negative for the *C. burnetii* case.

The GM antigen test was positive in three of four cases of documented aspergillosis on valve tissue and blood fungal PCR and in five of 59 BCNIE patients without detected aspergillosis, resulting in a sensitivity of 75%, specificity of 91.5%, positive predictive value of 37.5%, and negative predictive value of 98.2%.

Concerning IE complications, heart failure occurred in 18 (28.5%) patients. Embolization was detected in seven (11%) patients (four cerebrovascular, two pulmonary, and one peripheral). In five (7.9%) patients, relapse was documented (three with *S. aureus*, one with *Brucella*, and one with *Enterococcus faecalis*). Endocarditis-related mortality was recorded in eleven (17.4%) patients, attributed mainly to cardiovascular complications (seven patients), renal failure (three patients), and cerebrovascular complications (two patients).

Thus, using all diagnostic procedures, microbiological origin was defined in 59.6% (37/63) of BCNIE patients. This was attained by sequencing of blood PCR bands, serological assay, cardiac valve culture, and sequencing of valve PCR bands in 11/63, 15/63, 10/42, and 30/42 cases, respectively. In 26 (41.3%) BCNIE patients, the causative agent could not be detected. Therefore, the percentage of BCNIE with unidentified etiology was reduced from 67% (63/94) to 27.7% (26/94) through a combination of all diagnostic procedures utilized in our study.

## 4. Discussion

The use of molecular diagnostic techniques to diagnose IE has a short history. These methods are considered ground-breaking strategies for diagnosing IE [5,16] and have been introduced as a Duke major diagnostic criterion [16] because they can detect the causal pathogen in active endocarditis. One of the study’s goals was to assess the molecular and serological diagnosis of BCNIE. 

Our institute’s BCNIE proportion (67%) was regionally similar to that reported in two previous series, in which it accounted for 69.7% [14] and 69% [10] of definite IE cases. These rates are within the range of 15.5%–90% recorded in other developing countries [17,18,19,20,21]. However, BCNIE is relatively uncommon in Western countries, with rates of 9% in France [22] and 10% in Sweden [23]. This disparity could be explained by the free use of antibiotics in underdeveloped nations and the high occurrence of zoonotic illnesses caused by fastidious organisms. As a result, the implementation of an automated blood culture system at our institution is required for proper IE diagnosis.

In this study, the 16SrRNA and 18SrRNA PCR assay was positive in 17.5% of the blood samples, which was in line with the findings by Fournier et al. [24], who reported various PCR results depending on the tested sample. When applied to blood, bacterial 16S rRNA gene sequencing yielded only 35/257 positive samples, indicating a low sensitivity of 13.6%, which was in harmony with our bacterial 16S rRNA amplification result, which had a sensitivity of 12.7%. This low blood PCR sensitivity could be due to a smaller quantity of microorganisms in the blood due to previous antibiotic intake or, more likely, a failure of the traditional PCR technique to identify bacteria existing in a blood sample at a level below its sensitivity.

The sensitivity of histological evaluation of excised valvular material was 78.4%; comparable results with a sensitivity of 79% were documented in a previous study [13].

Zoonotic IE was diagnosed in 11% of patients with BCNIE using serologic and molecular procedures. Slightly higher rates were reported in studies conducted in Egypt and Algeria, in which zoonotic IE was observed in 12.8% and 15.6% of cases, respectively [17,25]. Four (6.3%) cases with *Brucella* spp., two (3.2%) cases with *Bartonella* spp., and one (1.2%) case with *C. burnetiid* were observed among our patients with zoonotic IE. According to previous studies, brucellosis is endemic in Egypt, with high seropositivity titers [13,26]; therefore, reinforcing brucellosis control methods in animals should be a top concern. Algeria had a higher rate of *Bartonella* (11.4%), followed by *Brucella* and *Coxiella* (3.3% each) [17], and Tunisia had a rate of *Bartonella* of 9.8% [27]. In contrast, *C. burnetii* was the most prevalent organism in the UK (12.7%), followed by *Bartonella* (1.1%) [28], and in France, *C. burnetii* represented 57.3% and *Bartonella* 19.2% of BCNIE cases [24]. Variations in socioeconomic conditions, animal exposure, veterinary care standards, and laboratory procedures may all contribute to these differences.

Of the seven cases of zoonotic IE, blood and valve tissue PCR correctly identified three of four *Brucella* cases and one of two *Bartonella* spp. cases but was negative for the *C. burnetii* case. Because these organisms are mostly found intracellularly in vegetations on affected heart valves, the positivity of each test may be influenced by the stage of disease [4]. This emphasizes the significance of serological diagnosis and valve PCR confirmation following surgical excision.

Many previous studies have documented fungal IE as a source of BCNIE [29,30]. Broad-range fungal 18SrRNA gene sequencing on blood, and valvular tissue detected four cases of *A. flavus* and one case of *Candida albicans* in our study. The GM antigen test was positive in four of five documented fungi on valve and blood fungal PCR, with a sensitivity of 75% and a specificity of 91.5%. Close sensitivity and specificity values of 83.3% and 84.2%, respectively, were recorded by Badiee et al. [31]. Many fungal and host factors influence the release of circulating galactomannan, including fungal strain, growth rate, underlying diseases, severity of the fungal infection, antifungal treatment, and age. Although the *Aspergillus* GM antigen assay can be a helpful supplementary test for establishing the diagnosis of invasive aspergillosis, its relevance in the setting of endocarditis has not been fully researched.

Recent studies employing a molecular approach for diagnosing BCNIE have found that valve material PCR exceeds traditional valve culture and microscopy [13,32,33]. This is consistent with our findings, which showed that valve PCR had a sensitivity of 71.4%, which was higher than that of valve culture (23.8%) and valve microscopy (11.9%). The limitations of valve culture in diagnosing the etiology of endocarditis could be due to antibiotic treatment prior to surgery.

### Study Limitations

We did not assess the existence of non-infective endocarditis in our analysis; however, it is likely that some of the 26 BCNIE patients with unknown etiology fall into this group. Furthermore, we did not explore all probable BCNIE etiological agents identified by serology (e.g., tests for *Mycoplasma* and *Chlamydia* were not performed). Another limitation was patient referral bias because the data were acquired from a single cardiac referral facility. In addition, the specificity, positive predictive value, and negative predictive value of 16S and 18S rRNA PCR could not be evaluated because almost all of the patients had definite BCNIE.

In conclusion, blood and valve PCR and sequencing assays are valuable techniques for the etiological diagnosis of BCNIE, especially in cases with previous antibiotic therapy. However, these tests should be used as part of a larger diagnostic strategy that includes serology, microscopy, and valve culture. The use of an automated blood culture system, as well as proper blood culture collection before ordering antibiotics, will guide the IE etiological diagnosis.

## Figures and Tables

**Table 1:** Compared positive results of sequencing of PCR positive valve section to valve culture and blood PCR.

Patient No.	Organisms Identified by Valve Broad-Range PCR and Direct Sequencing (Number)	Valve Culture	Blood Broad-Range PCR and Direct Sequencing	Histopathology
1	*Staphylococcus aureus*	Positive	Positive	typical IE
2	*Staphylococcus epidermidis*	Positive	Positive	typical IE
3	*Streptococcus mitis*	Negative	Negative	typical IE
4	*Brucella* spp.	Negative	Negative	typical IE
5	*Streptococcus mutans*	Negative	positive	typical IE
6	*Aspergillus flavus*	Negative	Negative	typical IE
7	*Streptococcus mitis*	It was positive for *Streptococci* spp.	Negative	typical IE
8	*Enterococcus faecalis*	It was positive for *Enterococci* spp.	Negative	typical IE
9	*Streptococcus oralis*	Negative	Negative	typical IE
10	*Streptococcus mitis*	Negative	Negative	typical IE
11	*Bartonella henselae*	Negative	Negative	typical IE
12	*Enterococcus faecalis*	Negative	Negative	typical IE
13	*Streptococcus agalactiae*	Negative	Positive	typical IE
14	*Streptococcus anginosus*	It was positive for *Viridans Streptococci*	Negative	typical IE
15	*Acinetobacter baumannii*	It was positive for *Pseudomonas aeruginosa*	Negative	typical IE
16	*Streptococcus mutans*	Negative	Negative	typical IE
17	*Haemophilus parainfluenzae*	Negative	Negative	typical IE
18	*Candida albicans* (1)	Positive	Positive	typical IE
19	*Staphylococcus aureus*	Negative	Negative	typical IE
20	*Staphylococcus epidermidis*	Negative	Negative	typical IE
21	*Streptococcus oralis*	Negative	Negative	typical IE
22	*Aspergillus flavus*	Negative	Negative	typical IE with dichotomously branched septate fungal hyphae on H&E
23	*Staphylococcus aureus*	Positive	Positive	typical IE
24	*Streptococcus mutans*	Negative	Negative	typical IE
25	*Staphylococcus aureus*	Negative	Negative	typical IE
26	*Brucella* spp.	Negative	Negative	typical IE
27	*Staphylococcus epidermidis*	Negative	Positive	typical IE
28	*Staphylococcus aureus*	Positive	Positive	typical IE
29	*Brucella* spp.	Negative	Negative	typical IE
30	*Aspergillus flavus*	Positive	Positive	typical IE with dichotomously branched septate fungal hyphae on H&E

**Table 2:** Pathogens isolated among all BCNIE patients using various diagnostic procedures.

Pathogen (Number)	Blood Broad-Range PCR and Direct Sequencing	Valve Culture	Valve Broad-Range PCR and Direct Sequencing	Serology
*Streptococcus* spp.(10)	**2**	**2**	**10**	**1 positive for GM**
*Staphylococcus aureus* (6)	**4**	**3**	**5**	
*Aspergillus flavus* (4)	**2**	**1**	**3**	**3 positive for GM**
*Staphylococcus epidermidis* (3)	**2**	**1**	**3**	**1 positive for GM**
*Brucella* spp. (4)	**-**	**-**	**3**	**4 positive for anti-*Brucella* antibodies**
*Enterococcus faecalis* (2)	**-**	**1**	**2**	
*Bartonella henselae* (2)	**-**	**-**	**1**	**2 positive for anti-*Bartonella henselae* antibodies**
*Acinetobacter baumannii* (1)	**-**	**Contamination (*Pseudomonas aeruginosa*)**	**1**	
*Haemophilus parainfluenzae* (1)	**-**	**-**	**1**	
*Candida albicans* (1)	**1**	**1**	**1**	**1 positive for GM**
*Coxiella burnetii* (1)	**-**	**-**	**-**	**1 positive for anti-*Coxiella burnetii* antibodies**

## Data Availability

This article contains all data produced or analyzed during this study. Accession numbers for some isolated organisms: *Staphylococcus aureus* (L37597), *Staphylococcus epidermidis* (L37605), *Streptococcus mitis* (D38482), *Streptococcus oralis* (X58308), *Brucella* spp. (AY594216).

## Abbreviations

BCNIE	Blood culture-negative infective endocarditis
IE	Infective endocarditis
PCR	Polymerase chain reaction
KOH	Potassium hydroxide
SDA	Sabouraud’s dextrose agar
GM	Aspergillus galactomannan antigen
